# Supplementary material for: Polyketide synthase gene clusters in the ericoid mycorrhizal fungus Oidiodendron maius: genome-wide prediction, expression profiling, and functional characterization of knockout mutants
Source: Front Plant Sci. 2026 Sep 16;17:1924430. doi: 10.3389/fpls.2026.1924430 (PMC13623903; doi:10.3389/fpls.2026.1924430)
Supplement: Supplementary file 1 [file SupplementaryFile1.zip › Supplementary Materials/Supplementary Folder 1.zip!/Oidma1_AssemblyScaffolds_Repeatmasked/knownclusterblast/cluster24/jgi.p_Oidma1_126236_mibig_hits.html]

| MIBiG Protein | Description | MIBiG Cluster | MiBiG Product | % ID | % Coverage | BLAST Score | E-value |
| --- | --- | --- | --- | --- | --- | --- | --- |
| EAU38977.1 | predicted\_protein | BGC0001122 | Isoflavipucine\_biosynthetic\_gene\_cluster | 33 | 103.929273084 | 271 | 1.7e-72 |
| BAE56594.1 |  | BGC0001123 | Aspirochlorine\_biosynthetic\_gene\_cluster | 30 | 96.6601178782 | 206 | 6.8e-53 |
| ACS68556.1 | major\_facilitator\_superfamily\_protein | BGC0001026 | NG-391\_biosynthetic\_gene\_cluster | 33 | 79.9607072692 | 205 | 2e-52 |
| ACZ66257.1 | APS11 | BGC0000304 | Apicidin\_biosynthetic\_gene\_cluster | 30 | 84.6758349705 | 177 | 3.4e-44 |
| AAB36607.1 | toxin\_pump | BGC0000370\_c2 | HC-toxin\_biosynthetic\_gene\_cluster | 31 | 85.4616895874 | 169 | 1.2e-41 |
| AGO50605.1 | transporter | BGC0000229 | Grincamycin\_biosynthetic\_gene\_cluster | 29 | 94.4990176817 | 163 | 5.1e-40 |
| BAV16993.1 | putative\_transporter | BGC0001384 | Saprolmycin\_biosynthetic\_gene\_cluster | 30 | 87.4263261297 | 156 | 8.1e-38 |
| WP\_052390548.1 | hypothetical\_protein | BGC0001308 | Simocyclinone\_D9\_/\_simocyclinone\_D10\_/\_simocyclinone\_D11 | 29 | 97.0530451866 | 156 | 1.1e-37 |
| AHV78251.1 | ResE | BGC0001246 | Trans-resorcylide\_biosynthetic\_gene\_cluster | 29 | 83.8899803536 | 152 | 1.2e-36 |
| ADM34144.1 | efflux\_pump | BGC0001084 | Notoamide\_/\_stephacidin\_biosynthetic\_gene\_cluster | 27 | 83.6935166994 | 150 | 4.5e-36 |
| AGC95323.1 | CurE | BGC0000045 | Dehydrocurvularin\_biosynthetic\_gene\_cluster | 28 | 80.7465618861 | 144 | 2.4e-34 |
| AEI98661.1 | CtcR | BGC0000209 | Chlortetracycline\_biosynthetic\_gene\_cluster | 30 | 81.3359528487 | 137 | 3.9e-32 |
| EAA65599.1 | hypothetical\_protein | BGC0000022 | Asperfuranone\_biosynthetic\_gene\_cluster | 29 | 68.7622789784 | 131 | 2.1e-30 |
| WP\_016325506.1 | hypothetical\_protein | BGC0001283 | Arsenopolyketides\_biosynthetic\_gene\_cluster | 27 | 96.463654224 | 123 | 7.6e-28 |
| CAH10178.1 | ChaT1\_protein | BGC0000206\_c2 | Chartreusin\_biosynthetic\_gene\_cluster | 24 | 86.6404715128 | 115 | 1.6e-25 |
| AJI44168.1 | hypothetical\_protein | BGC0001193 | Thiolutin\_biosynthetic\_gene\_cluster | 30 | 83.1041257367 | 115 | 2.1e-25 |
| ADO85573.1 | PntG | BGC0000653 | Pentalenolactone\_biosynthetic\_gene\_cluster | 34 | 32.8094302554 | 75 | 1.8e-13 |
| AAM54113.1 | bicyclomycin\_resistant\_protein | BGC0000020 | Ansamitocin\_biosynthetic\_gene\_cluster | 30 | 32.2200392927 | 64 | 4.2e-10 |
| BAM73629.1 | putative\_bagremycin\_transporter | BGC0000889 | Bagremycin\_biosynthetic\_gene\_cluster | 28 | 41.6502946955 | 62 | 1.6e-09 |
| BAG21071.1 | putative\_grixazone\_transporter | BGC0000662 | Grixazone\_biosynthetic\_gene\_cluster | 26 | 57.3673870334 | 50 | 8.2e-06 |
